# Novel transplant of combined platelet-rich fibrin Releasate and bone marrow stem cells prevent bone loss in Ovariectomized osteoporotic mice

**DOI:** 10.1186/s12891-020-03549-y

**Published:** 2020-08-08

**Authors:** Chin-Chean Wong, Jeng-Hao Liao, Shi-Yuan Sheu, Po-Yu Lin, Chih-Hwa Chen, Tzong-Fu Kuo

**Affiliations:** 1 Department of Orthopedics, Shuang Ho Hospital, Taipei Medical University, New Taipei City, 23561 Taiwan; 2 Department of Orthopedics, School of Medicine, College of Medicine, Taipei Medical University, Taipei, 11031 Taiwan; 3 Research Center of Biomedical Devices, Taipei Medical University, Taipei, 11031 Taiwan; 4 International Ph.D. Program for Cell Therapy and Regenerative Medicine, College of Medicine, Taipei Medical University, Taipei, 11031 Taiwan; 5 Non-invasive Cancer Therapy Research Institute of Taiwan, Taipei, 10489 Taiwan; 6 School of Veterinary Medicine, National Taiwan University, Taipei, 10617 Taiwan; 7 School of Chinese Medicine for Post-Baccalaureate, I-Shou University, Kaohsiung, 84001 Taiwan; 8 Department of Chinese Medicine, E-Da Cancer Hospital, Kaohsiung, 84001 Taiwan; 9 School of Biomedical Engineering, College of Biomedical Engineering, Taipei Medical University, Taipei, 11031 Taiwan; 10 School of Medicine, College of Medicine, Taipei Medical University, Taipei, 11031 Taiwan; 11 Department of Post-Baccalaureate Veterinary Medicine, Asia University, Taichung, 41354 Taiwan

**Keywords:** Bone marrow, Osteoporosis, Platelet-rich fibrin, Releasates, Stem cell

## Abstract

**Background:**

Osteoporosis is a metabolic bone disorder characterized by deterioration in the quantity and quality of bone tissue, with a consequent increase susceptibility to fracture.

**Methods:**

In this study, we sought to determine the efficacy of platelet-rich fibrin releasates (PRFr) in augmenting the therapeutic effects of stem cell-based therapy in treating osteoporotic bone disorder. An osteoporosis mouse model was established through bilateral ovariectomy on 12-week-old female ICR (Institute of Cancer Research) mice. Eight weeks postoperatively, the ovariectomized (OVX) mice were left untreated (control) or injected with PRFr, bone marrow stem cells (BMSCs), or the combination of BMSCs and PRFr. Two different injection (single versus quadruple) dosages were tested to investigate the accumulative effects of BMSCS and PRFr on bone quality.

Eight weeks after injection, the changes in tibial microstructural profiles included the percentage of bone volume versus total tissue volume (BV/TV, %), bone mineral density (BMD, g/cm3), trabecular number (Tb.N, number/mm), and trabecular separation (Tb.Sp, mm) and bony histology were analyzed.

**Results:**

Postmenopausal osteoporosis model was successfully established in OVX mice, evidenced by reduced BMD, decreased BV/TV, lower Tb.N but increased Tb.Sp. Eight weeks after injection, there was no significant change to BMD and bone trabeculae could be detected in mice that received single-injection regimen. In contrast, in mice which received 4 doses of combined PRFr and BMSCs, the BMD, BV/TV, and TB.N increased, and the TB.Sp decreased significantly compared to untreated OVX mice. Moreover, the histological analysis showed the trabecular spacing become narrower in OVX-mice treated with quadruple injection of BMSCs and combined PRFr and BMSCs than untreated control.

**Conclusion:**

The systemic administration of combined BMSCs and PRFr protected against OVX-induced bone mass loss in mice. Moreover, the improvement of bony profile scores in quadruple-injection group is better than the single-injection group, probably through the increase in effect size of cells and growth factors. Our data also revealed the combination therapy of BMSCs and PRFr has better effect in enhancing osteogenesis, which may provide insight for the development of a novel therapeutic strategy in osteoporosis treatment.

## Background

Osteoporosis is a systemic skeletal disease characterized by low bone mineral density (BMD) and impaired bone microarchitecture [1]. As a consequence, an increased risk of fractures, in particular hip, spine, and wrist would occur in osteoporotic patients who sustained low energy injury [2]. Throughout life, bones are in dynamic equilibrium, meaning that they are continuously remodeled by osteoclast-mediated bone resorption and osteoblast-mediated bone formation [3]. However, the disruption of equilibrium of bone remodeling would happen when the bone-forming osteoblasts activity is surpassed by osteoclast-mediated bone resorption thereby contributes to the pathogenesis in osteoporosis [4–7]. The current medications for osteoporosis can be categorized into either anti-resorptive drugs (i.e., bisphosphonates, estrogen agonist/ antagonists, estrogens, calcitonin, and denosumab) or anabolic agent (i.e., teriparatide) [1]. In recent years, anti-sclerostin monoclonal antibody has been demonstrated to increase bone formation thus with the potential to improve clinical outcomes in osteoporotic patients [8, 9]. Although current evidences have shown encouraging results of both types of drugs in improving BMD and reducing fracture risk, there are growing concerns about devastating drug-related complications such as osteonecrosis of jaw and atypical femoral fractures [10–12].

Osteogenesis and bone remodeling are complex processes that involve the interactions between distinct cell populations, not only the osteoblastic and osteoclastic lineages, but also an interplay between progenitor stem cells, hematopoietic and immune cells [13]. Despite the complex nature of the pathogenesis of osteoporosis, osteoblast undoubtedly play a key role in disease’s pathogenesis via participating in the bone forming process. On the other hand, accumulating evidence has revealed an association of a stem cell population, derived from bone marrow in bone maintenance and remodeling [13]. Under physiological conditions, continuous and harmonious bone remodeling is maintained by a well-orchestrated osteoclast-osteoblast coupling and the balance between the osteogenic and adipogenic abilities of the stem cells. Postmenopausal estrogen deficiency breaks the balance of osteoclastogenesis and osteoblastogenesis, resulting in progressive bone loss [3, 14, 15]. Additionally, the dysfunction of bone marrow stem cells (BMSCs) attributed to menopause, aging or ovariectomy would therefore impair their proliferative response and osteogenic differentiation abilities [16], resulting in bone mass loss and osteoporosis [17]. Moreover, the increase in the number of adipocytes would subsequently induced further apoptosis in osteoblasts and promoted the proliferation and differentiation of osteoclasts, resulting in an increase in osteoclastic resorption and overall bone loss [18, 19]. From this standpoint, it would be plausible to develop the stem cell transplantation and cell-based therapies as a therapeutic strategy to provide the osteoblastic niche to reverse the shift towards bone resorption in osteoporosis.

Platelet-rich plasma (PRP) is a platelet concentrate derived from autologous blood and was effective in promoting proliferation and osteogenic activity of BMSCs and osteoblasts [20–22]. Unlike PRP, platelet-rich fibrin (PRF) is a gel-like platelet concentrate yielded by natural polymerization process during centrifugation without additives such as thrombin, calcium chloride or anticoagulants [23, 24]. Its fibrin microarchitecture is beneficial for the containment and sustained release of growth factors over time [25]. Furthermore, the PRF releasates (PRFr) is a supernatant rich in growth factors which is separated from PRF gel through the process of quiescence and centrifugation. In previous report, PRFr could induce human mesenchymal stem cells osteogenic differentiation as characterized by the formation of calcium ossificates, increased alkaline phosphatase activity and upregulation of osteogenic markers [26]. However, the effects of PRFr and BMSCs or their combinatory use in osteoporotic treatment remained largely unknown and need to be elucidated.

In this study, we intend to explore the potential of the novel application of BMSCs and PRFr in preventing bone loss using osteoporotic mouse model. We hypothesize that the combination therapy of BMSCs and PRFr would enhance osteogenesis, thereby favoring bone formation and ameliorate osteoporosis. Micro-computer tomography (micro-CT) imaging and histology were employed to analyze bony profile changes before and after treatments. Moreover, the dose-dependent effect of each therapeutic is examined to help determining optimal treatment regimen in future clinical application.

## Methods

### Study design and ethics statement

All procedures of platelet-rich fibrin and bone marrow stem cells isolation and surgery on experimental animals were carried out according to the guide for the Care and Use of Laboratory Animals and was approved by the Institutional Animal Care and Use Committee (IACUC approval: NTU-102-EL-82; NTU-102-EL-91).

### Harvest and cultivation of bone marrow stem cells

Bone marrow aspirates were obtained aseptically from mouse femurs. Bone marrow specimen was collected from the disposed aspirates using a 10 mL syringe. The aspirates were immediately mixed with 0.5 mL of sodium-heparin (10,000 U/mL) and diluted in equal volume of phosphate-buffered saline (PBS). The cell suspension was then fractionated on a Lymphoprep (Fisher Scientific, Goteborg, Sweden) and centrifuged at 400 *g* for 30 min. The interface fraction enriched with bone marrow stem cells (BMSC) was collected and plated onto a 10 cm dish containing 10 mL of α Modified Eagles Medium (αMEM) containing 10% of fetal bovine serum (FBS) (Gibco, Paisley, UK), and 1X P/S/A (penicillin/ streptomycin/ fungizone). After washing out non-adherent hematopoietic cells, the adherent BMSCs were cultured in 5% CO_2_ at 37 °C with medium changed every 3 ~ 4 days. When the cells reached 80% confluence, they were trypsinized and passaged into new 10-cm dishes at a cell density of 5 × 10^5^ cells/ dish. The cells were sub-cultured till passage 2 (P2). P2 cells were then seeded at a cell density of 6.5 × 10 ^3^ cells/ cm^2^ for further in vitro tests.

### Flow cytometry analysis

BMSCs were fixed with ethanol overnight at − 20 °C. Aliquots of 5 × 10^5^ cells were incubated with each of the fluorochrome-conjugated antibodies against a panel of cell surface markers, including CD 34 (BD 553731, Biosciences, USA), CD 45 (AB 10558, Abcam, USA), CD 44 (AB 119335, Abcam, USA) and CD 90 (BD 554895, BD Biosciences, USA) at 4 C°. Cells were resuspended in Con’s tube (BD) containing 200 uL of PBS/ 1% bovine serum albumin and analyzed by flow cytometry using the FACScan system (Becton Dickinson, USA).

### Preparation of platelet-rich fibrin releasates

A laboratory mouse has a circulating blood volume of about 1.5–2.5 mL (6–8% of the body weight) [27], thus it would be difficult to obtain sufficient amount of mouse blood for experimental use. Since rabbits are of the order lagomorphs similar to rodents, thus large lot sizes available from pooled rabbit donors may be an alternative blood source [28]. In this study, blood samples were collected from the New Zealand White rabbits (mean weight 3 ~ 3.5 kg) under inhalational anesthesia with isoflurane. The PRF gel was prepared using the technique described by Choukroun et al. [23]. After adequate skin cleaning, disinfection and sterilization, 8 mL of venous blood was harvested from rabbits in sterile tubes without anticoagulant supplement, and then centrifuged at 400 g for 10 min in a DSC-200A-2 table top centrifuge (Digisystem, Laboratory Instruments Inc., New Taipei City, Taiwan). The acquired PRF gel was located between the red blood cells and the acellular plasma. The PRF gel was then transferred into 15 mL sterile centrifuge tube and was allowed to stand quiescence for 5 h to allow PRF releasates (PRFr) formation. According to Su et al. study, the PRFr collected at 5 h contains notably higher concentration of growth factors which would be beneficial for bone formation [29]. Later, the PRF gel was centrifuged at 3000 g for 10 min and the resultant PRFr located in supernatant fraction was carefully aspirated and stored at − 20 °C until use.

### Cytokine assay of platelet-rich fibrin releasate

The PRFr derived from blood samples of five different rabbits were collected for quantitation of cytokine content. The concentrations of cytokine including platelet-derived growth factor (PDGF)-AA and BB, transforming-growth factor-β (TGF-β), and bone morphogenetic protein-2 (BMP-2) were measured using commercially available bead-based sandwich immune-assay kits ((PDGF AA & BB: Bioassay Technology Laboratory, Shanghai, China; TGF-β: Abbkine, CA, USA; BMP-2: Cloud-clone, TA, USA). Standards and samples were assayed in five replicates and mean values were recorded. The detection limit was 5, 2, 15 and 15.6 pg/mL for PDGF-AA, PDGF-BB, TGF-β and BMP-2 respectively.

### Osteogenic differentiation assay

The osteogenic potential of BMSCs was assessed by osteogenic differentiation assay. Osteogenic induction medium was prepared using αMEM medium supplemented with 15% FBS, 50 mg/mL L-ascorbate-2-phosphate, 10^− 7^ M dexamethasone and 10 mM β-glyceralphosphate. The P2 BMSCs were suspended in αMEM medium at a density of 1 × 10^4^ cells/mL per well and loaded on 24-well plate. After 24 h, the medium was removed and 1 mL of αMEM medium or osteogenic medium were added, respectively. The medium was changed every 3 days, for a total culture period of 14 days. To detect calcium deposition, the differentiated BMSCs were fixed with 4% formaldehyde at room temperature for 30 min and rinsed rapidly with distilled water. Then, 1 mL of pH 4.2 Alizarin Red S solution (Sigma, St. Louis, USA) was added to cover cell surface for 5 min, followed by washing thoroughly with distilled water. The calcium deposits exhibited as orange red sediments on the cell surface and were recorded microscopically. For quantitative analysis, the intensity of calcium deposition in each group was counted with image analysis software (Image J, Version 1.52a, National Health Institute) in triplicate.

### Establishment of mouse osteoporotic model via bilateral ovariectomy

Postmenopausal osteoporosis mouse model was established through bilateral ovariectomy (OVX). The female ICR mice (BioLASCO Taiwan Co., Ltd., Taipei, Taiwan) were maintained in polycarbonate cages at 21 ± 2 °C with a 12 h’ light/dark cycle and given access to food and water ad libitum for 4 weeks as an adaptation period. Before surgery, the mice were placed under general anesthesia consisting of an intramuscular injection (0.25 mL/kg) with 1:1 mixture of Zoletil®50 (Virbac Laboratories, Carros, France) and xylazine (Balanzine®, Health-Tech Pharmaceutical Co., Taiwan). The animals were infiltrated with 1% lidocaine to the incision site to reduce the operative pain. A 1-cm longitudinal incision was made at the intersection which was 0.5 cm from the central spine and 1 cm below the lower rib edge. In control group (non-OVX), the mice had received sham surgery without ovary removal. For OVX-mice, the ovarian fat pad was lifted, and the bilateral ovaries were exteriorized and carefully removed followed by suture ligations to minimize bleeding. The abdominal muscles were then closed using an absorbable suture (Vicryl 3.0, ETHICON Inc., Somerville, NJ, USA). The OVX mice were then observed twice a day for a total period of 14 days.

### Micro-computed tomography (micro-CT)

Eight weeks postoperatively, micro-CT was used to evaluate the changes in bone mineral density (BMD) and trabecular bone morphology between non-OVX and OVX mice. The protocol of micro-CT used in this study was described as follow. The left tibia of animal was harvested and was placed in a custom jig with water and scanned with a SkyScan 1176 Micro CT scanner (SkyScan, Kontich, Belgium) operating at 40 kV, 600 μA, 0.3 μ of rotation step, 0.5 mm A1 filter and 9 mm/pixel of scan resolution. The image slices were reconstructed using the NRecon (v. 1.4.4, Skyscan) software system. The three dimensional (3D) parameters of bone microarchitecture were calculated using CTAn (v.17.0.0, Skyscan) software. For trabecular bone, the proximal tibia was selected for analysis within a conforming volume of interest commencing at the growth plate and extending a further longitudinal distance of 0.5–1.5 mm in the proximal direction to assess site-specific responses to ovariectomy (Fig. 2a). Microstructural measurements included the percentage of bone volume versus total tissue volume (BV/TV, %), bone mineral density (BMD, g/cm3), trabecular number (Tb.N, /mm), and trabecular separation (Tb.Sp, mm).

### In vivo intravenous administration of BMSCs and PRFr

Forty-two OVX mice were used for experiment and were allocated into 7 groups. The OVX mice which received no treatment served as control. To evaluate the accumulative effects of PRFr and BMSCs on bone mass, two different treatment groups were compared, known as the single-injection group or quadruple-injection group. PRFr and cell suspension were administrated through tail vein injection using a 26-gauge needle. Basically, in single-injection group, mice received single dose of either 0.6 mL of PRFr, 3 × 10^5^ BMSCs or 0.6 mL of PRFr containing 3 × 10^5^ of BMSCs. In quadruple-injection group, mice received injection of either 0.6 mL of PRFr, 3 × 10^5^ of BMSCs or 0.6 mL of PRFr containing 3 × 10^5^ of BMSCs once a week, for four consecutive weeks. Eight weeks after treatment, the mice were euthanized by carbon dioxide inhalation with flow rate 3 l/minute. For all animals, the carbon dioxide inhalation was continued until 1 min after breathing stops. The euthanized animals were then subjected for micro-CT analysis followed the protocol as previously described.

### Histological analysis

For histological evaluation, the tibia specimens were decalcified and fixed in 10% buffered formalin for at least 48 h before routine processing. The samples were then embedded in paraffin and the blocks were cut into serial 5-μm thick sections and stained with hematoxylin and eosin (H&E). The specimen sections were examined under light microscopy (Leica, Japan) for evaluation of trabecular changes.

### Statistical analysis

For the quantitative assay, each data point was derived from three independent experiments or an experiment of quadruplicate assay and was presented as mean with standard deviation. All analyses were performed using GraphPad Prism 8.0 analytic software. Statistical significance was set at a *p* value of < 0.05. The data were analyzed using student t-test, and one-way ANOVA followed by post hoc scheffe test and multiple comparisons Dunnett test. The statistical significances among the experimental groups were indicated with asterisks. Groups labeled with asterisk superscript letters, indicate that the statistic difference between the two groups had the *p* value less than 0.05, and was considered significantly different.

## Results

### Characterization and osteogenic potential of BMSCs

**Fig. 1:**
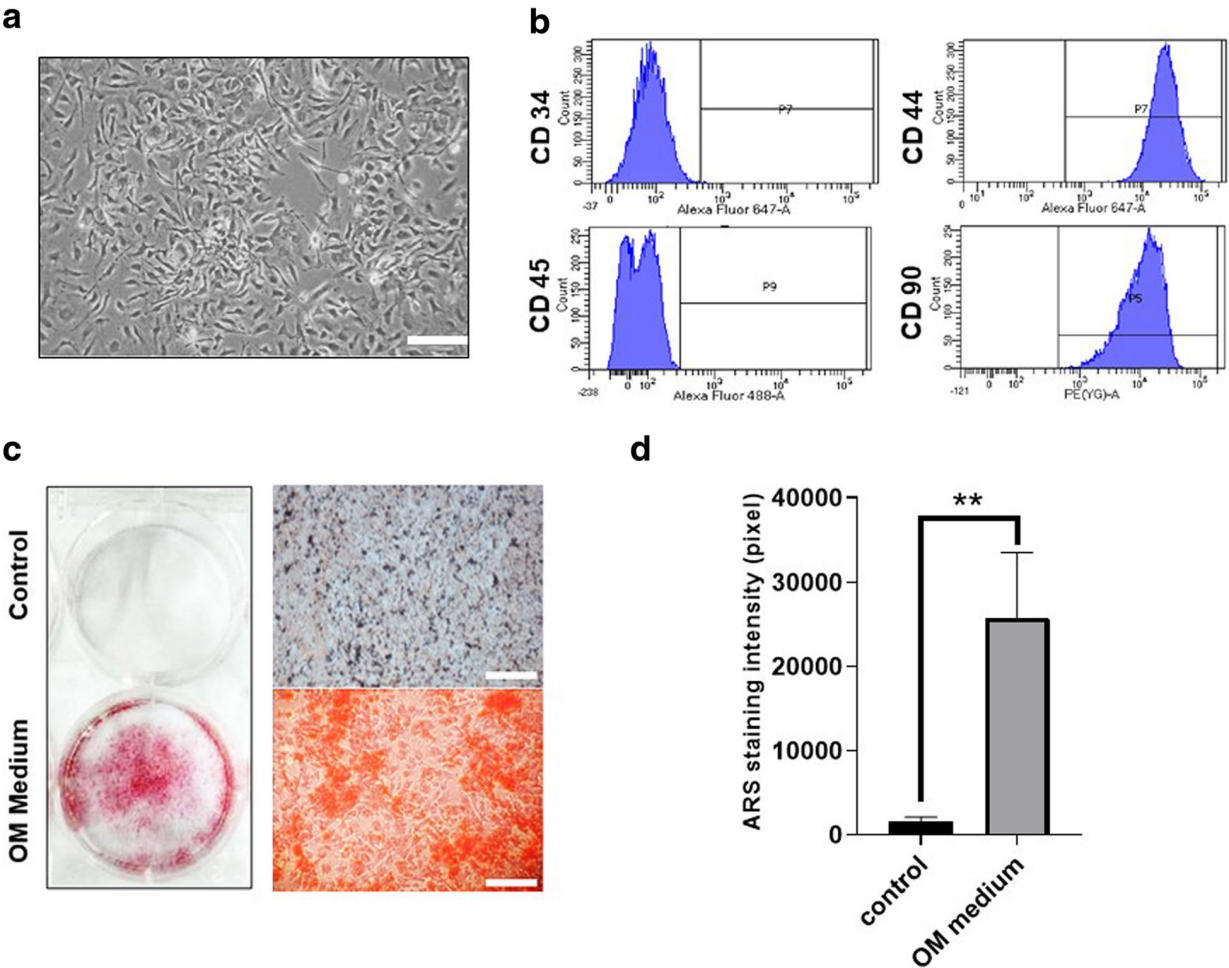
Cell morphology, surface marker expressions and osteogenic differentiation potential of bone marrow stem cells (BMSCs). **a** Colony forming unit is shown under bright field observation in the passage 0 BMSC culture. **b** For flow cytometry analysis, the BMSCs were found to exhibit a phenotype of CD44+ CD90+ CD34- CD45-. **c** Alizarin Red S (ARS) staining of BMSCs cultured in standard medium (upper panel) or osteogenic differentiation medium (lower panel) for 14 days. **d** Quantitation of ARS staining intensity. Scale bar- 100 μm. The bars show the mean ± SD (*n* = 3) of each group. **P < .01

The BMSCs were cultivated, expanded and cell morphology was closely monitored. Figure 1a show the colony forming units in BMSC primary culture with fibroblast-like morphology. The surface markers of BMSCs were characterized by flow cytometry analysis as shown in Fig. 1b. A phenotype of CD44^+^ CD90^+^ CD34^−^ CD45^−^ cell surface markers was identified in the P2 BMSCs. To examine the osteogenic capacity of the cells used in this study, the P2 BMSCs were further treated with osteogenic medium for 14 days to test their ability towards osteoblasts. Figure 1c showed that after 14 days of osteogenic induction, the culture dish showed significant reddish precipitation after alizarin red S (ARS) staining, representing the mineralization of the culture. Figure 1d showed the quantitative analysis of ARS staining intensity between two groups.

### Cytokine quantification of PRFr

In general, 0.2 mL of PRFr could be retrieved from 1 mL of whole venous blood after the PRF was left quiescence for 5 h. As shown in Table 1, the average concentration of PDGF-AA and BB is 388.88 ± 51.71 ng/L and 174.77 ± 23.76 ng/L, respectively. For TGF-β, the average concentration is 53.27 ± 3.46 ng/L. For BMP-2, the average concentration is 94.15 ± 10.9 pg/mL.

**Table 1:** Cytokine concentrations of Platelet-rich fibrin releasates derived from different rabbits

Cytokine Concentration (*n* = 5)	PDGF-AA (ng/L)	PDGF-BB (ng/L)	TGF-β (ng/L)	BMP-2 (pg/mL)
**Mean ± SD**	388.88 ± 51.71	174.77 ± 23.76	53.27 ± 3.46	94.15 ± 10.9

### Changes of BMD and bony trabecular in ovariectomized mice

**Fig. 2:**
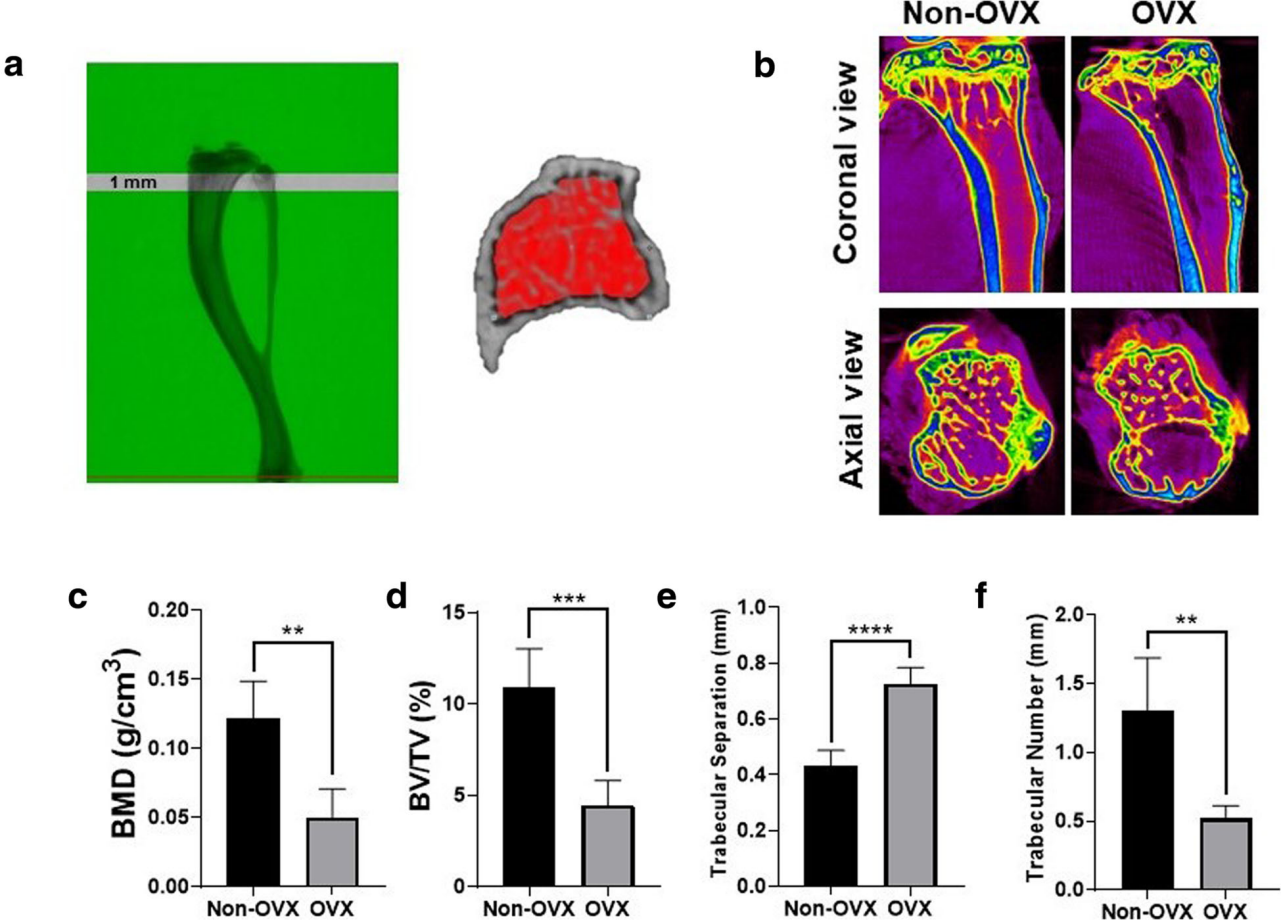
**a** Schematic representation of the trabecular bone area using for micro-computed tomography (micro-CT) analysis. The proximal tibia was selected for analysis within a conforming volume of interest commencing at the growth plate and extending a further longitudinal distance of 0.5 to 1.5 mm in the proximal direction to assess site-specific responses to ovariectomized and treatments. **b** Comparison of coronal and axial view of micro-CT images of non-ovariectomized (Non-OVX) and ovariectomized (OVX) mice. **c** Micro-CT analysis of bone marrow density (BMD), bone volume versus total tissue volume (BV/TV, %), trabecular number (Tb.N), and trabecular separation (Tb.Sp) in non-OVX and OVX mice eight weeks after surgery. The bars show the mean ± SD (*n* = 6) of each group. **P < .01; ***P < .001; ****P < .0001

Figure 2b showed the significant morphological differences in bony profile of non-OVX and OVX mice as demonstrated by micro-CT analysis, characterized by a reduction in bony trabecular and increase in trabecular separation seen in OVX mice. Moreover, compared to non-OVX control, OVX mice exhibited reduced BMDs (0.12 ± 0.03 g/cm^3^ vs. 0.05 ± 0.02 g/cm^3^; *p* < 0.01), decreased BV/TV (10.89 ± 2.13% vs. 4.46 ± 1.35%; *p* < 0.001), and lower Tb.N (1.31 ± 0.38 mm vs. 0.52 ± 0.1 mm; *p* < 0.001), but increased Tb.Sp (0.43 ± 0.05 vs. 0.73 ± 0.06 mm; *p* < 0.0001), 8 weeks after surgery (Fig. 2c-f). The results indicate that the osteoporosis model has been successfully established.

### Micro-CT analysis of BMD and bony trabecular morphology

**Fig. 3:**
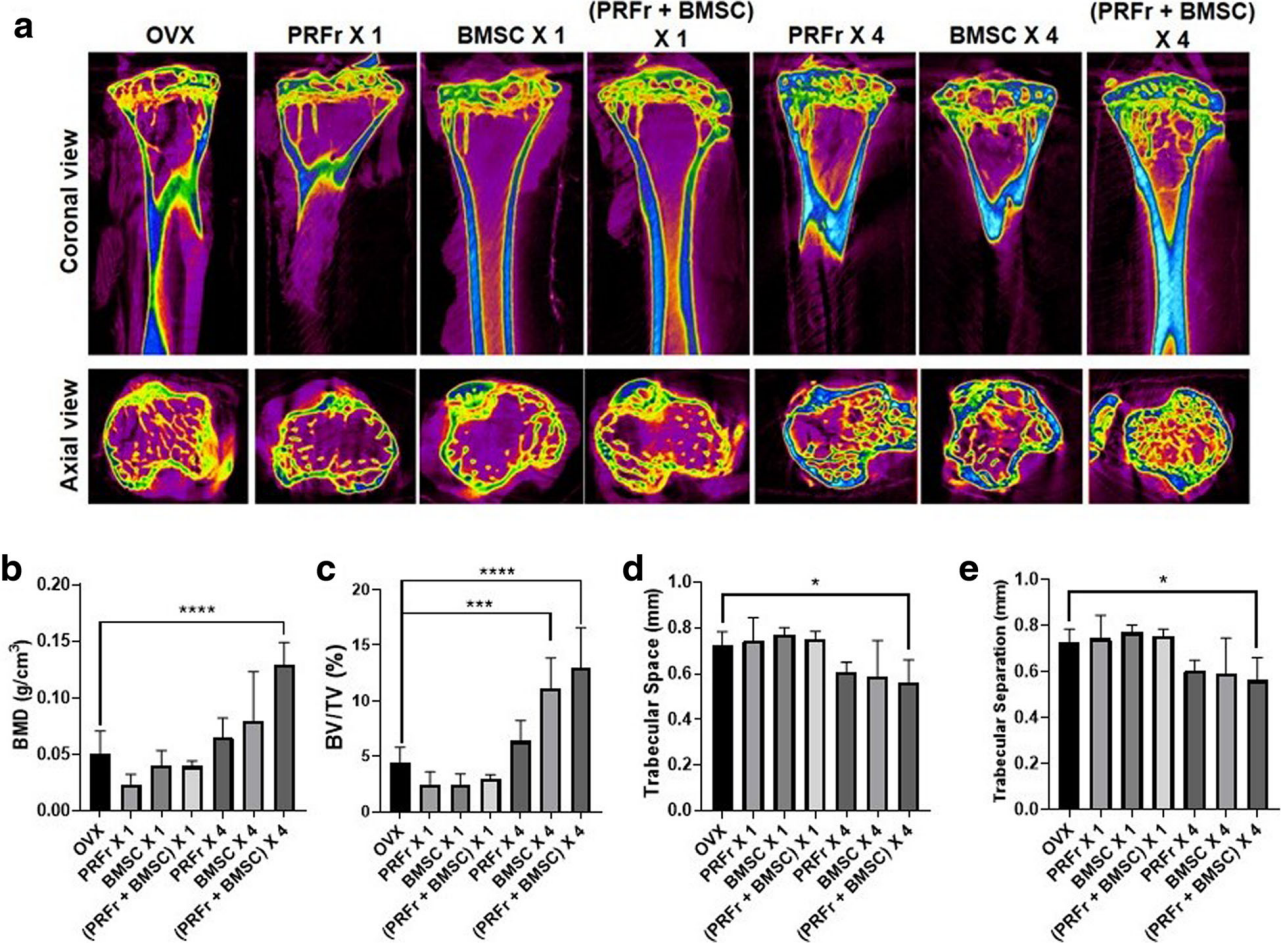
Micro-computed tomography (micro-CT) images of ovariectomized mice in untreated control (OVX), or experimental groups which received single/quadruple injection of either platelet-rich fibrin releasate (PRFr), bone marrow stem cells (BMSC) or in combination therapy (PRFr + BMSC). Comparison of coronal and axial view of micro-CT images in different groups. **b**-**e** The bone marrow density (BMD), bone volume versus total tissue volume (BV/TV, %), trabecular number (Tb.N), and trabecular separation (Tb.Sp) in each group of mice were evaluated 8-weeks after injection. The bars show the mean ± SD (*n* = 6) of each group. *P < .05; ***P < .001; ****P < .0001

**Fig. 4:**
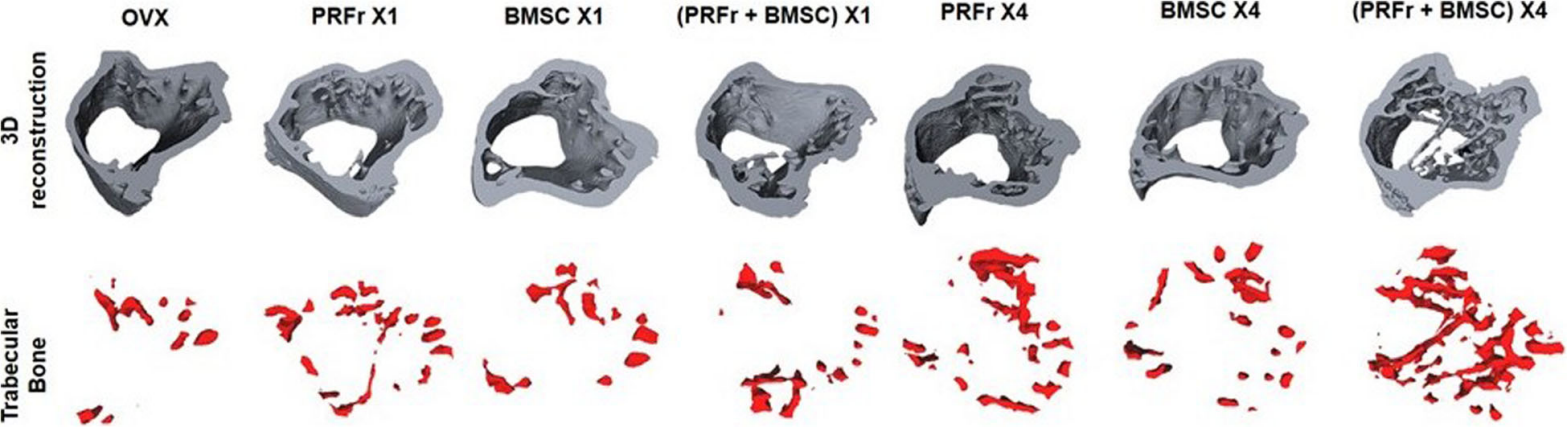
Three-dimensional (3D) reconstructed images of ovariectomized mice in untreated control (OVX), or experimental groups which received single/quadruple injection of either platelet-rich fibrin releasate (PRFr), bone marrow stem cells (BMSC) or in combination therapy (PRFr + BMSC) (Upper panel). The red region in the lower panel represents the 3D scope of the newly formed trabecular bone in the proximal tibial

Figure 3a showed the micro-CT images of tibial bone of OVX mice which were left untreated (*n* = 6) or received treatments with either single/quadruple injection of PRFr (*n* = 6), BMSCs (*n* = 6), or combined PRFr and BMSCs (*n* = 6). The trabecular architecture and new bone formation were demonstrated on coronal view and axial view in each group. On both views, OVX mice which received quadruple-injection regimen showed significantly more new bone formation at 8 weeks’ post-treatment. Quantitatively, compared to untreated OVX mice, there was no significant changes to BMD and bone trabeculae could be detected in mice that received single-injection regimen, regardless the content of injection. On the other hand, the results also showed that there was a trend demonstrating an increased bony profile occurred in quadruple-injection treatment groups. Additionally, in mice which received 4 doses of combined PRFr and BMSCs, the BMD, BV/TV, and TB.N increased, and the TB.Sp decreased significantly compared to untreated OVX mice (Fig. 3b-e). 3D reconstructed images showed that the trabecular bone and new bone formation were significantly more abundant in quadruple injection groups compared to untreated control (Fig. 4).

### Histological analysis

**Fig. 5:**
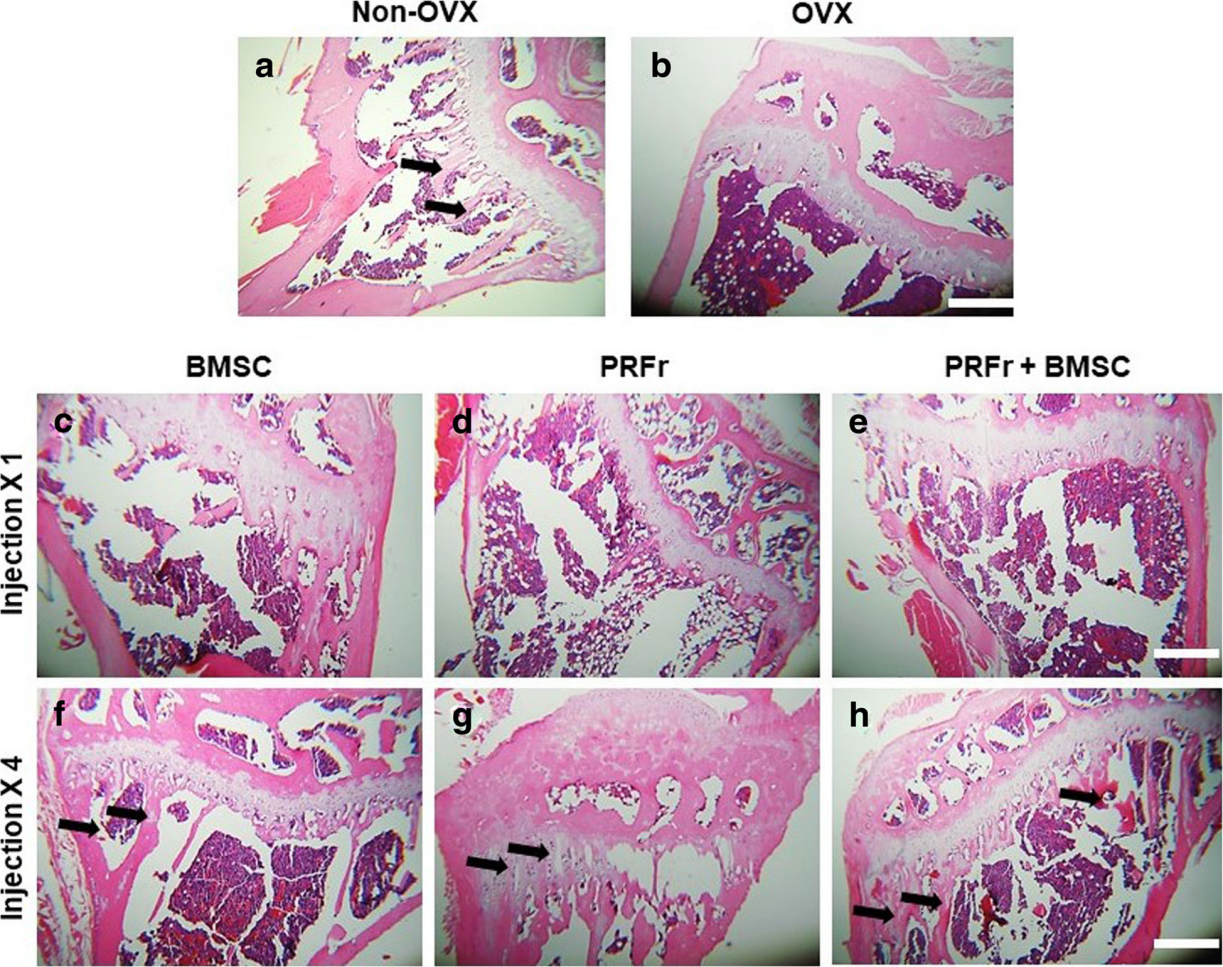
Histological sections of proximal tibial bony architecture in non-ovariectomized (Non-OVX) mice (**a**) and ovariectomized (OVX) mice (**b**) stained by hematoxylin and eosin (H&E). **c**-**e** Proximal tibial sections from mice received single/quadruple injection of either platelet-rich fibrin releasate (PRFr), bone marrow stem cells (BMSC) or in combination therapy (PRFr + BMSC). The black arrows indicate newly formed bony trabeculae. Scale bar- 2.5 mm

Figure 5 showed the histological images of the tibial bone in all groups stained by H&E. The results showed that the number of bony trabeculae decreased significantly in OVX mice compared to non-OVX mice. No significant changes in trabecular number was found in single-injection groups. In contrast, the trabecular spacing become narrower in OVX-mice treated with quadruple injection of BMSCs and combined PRFr and BMSCs than untreated control. Moreover, the trabecular conjunction points were obviously increased in OVX groups treated with quadruple injection of combined PRFr and BMSCs.

## Discussion

Fragile fractures resulting from osteoporosis are a major cause of morbidity and mortality in elderly patients [30]. In osteoporotic bone, the proportion of fat content in the bone marrow increased remarkably and was shown to be closely linked to a decreased in BMD [15]. The apparent implication of this clinical phenomenon is an inverse relationship between bone marrow adipogenesis and osteogenesis coupling. This important observation was further reinforced by evidence that an increasing number of adipocytes subsequently suppressed bone marrow stem cells (BMSCs) osteogenesis, leading to increased bone resorption and thus overall bone loss [14, 15, 31, 32]. Based on these findings, many studies have emphasized the pivotal role of stem cells in osteoporosis pathogenesis since their osteogenic ability would directly affect in vivo osteogenesis and bone matrix mineralization. From this standpoint, developing stem cell-based therapy to overcome the substantial decline in the number and function of osteogenic cells by means of stem cell transplantation emerge a major research focus in osteoporotic treatment.

The aims of this present study are to evaluate the therapeutic efficacy of BMSCs and PRFr in preventing bone loss in a well-established osteoporotic mouse model. In our previous studies, PRF displayed pronounced stimulatory effects on cell proliferation, chemotaxis, and differentiation [33–35]. Moreover, most of the studies showing a positive role for platelet concentrate in bone regeneration both in vitro and in vivo [15, 20, 21, 26, 36, 37]. Nonetheless, limited reports are available exploring the efficacy of isolated use of BMSCs and PRFr treatments in an osteoporotic model or their combination use in preventing bone loss. In this study, we found that systemically administered combined BMSCs and PRFr could effectively prevent ovariectomy-induced bone mass attenuation. Our data revealed that the combined use of BMSCs and PRFr has significant better effect in enhancing bone formation, which may translate into a plausible therapeutic strategy in osteoporosis treatments.

PRF is a second-generation platelet concentrate produced from autologous blood obtained immediately after single-spin centrifugation [24]. Rapid activation of the coagulation cascade and synthesis of thrombin take place when platelets get contact with glass particles of test tube. A laboratory mouse has a circulating blood volume of only 1.5 to 2.5 mL, therefore it is unrealistic and difficult to obtain sufficient volume of mouse blood for PRFr preparation [27]. In this study, xenogeneic PRFr obtained from rabbit’s donor was prepared through quiescence and double centrifugation. Through this process, only the bioactive molecules released by activated platelets were collected, thereby preserving the bioactivity of PRFr and minimizing the immunogenicity of rabbit derived PRFr. When PRFr was implanted in vivo, it could serve as the molecular modulator of osteogenesis because PRFr contains a wide spectrum of growth factors such as PDGF, TGF-β, BMP-2 and many others that are able to modulate the regenerative process [23, 38–41]. Moreover, many researches have highlighted the supportive role of platelet concentrate in osteogenesis by promoting cell proliferation, chemotaxis, osteogenic differentiation, and extracellular mineralization [15, 21, 26, 36]. In Parsons et al. study, platelet concentrate enhances cellular growth of human mesenchymal stem cells and osteogenic marker expressions [21]. Zhang et al. also demonstrated that treatment of rabbit bone marrow mesenchymal stem cells with exogenous platelet-rich plasma promoted osteogenic gene expressions, including alkaline phosphatase (ALP), Osteocalcin (OCN) and runt-related transcription factor-2 (RUNX2) [42]. More importantly, PRP could not only promote osteogenesis but also inhibit adipogenesis of pre-adipocytes. The overall enhanced osteogenesis by PRP treatment was observed in Liu et al. study via the simultaneous up-regulation of osteogenic markers (OCN, RUNX2 and osteopontin) and down-regulation of adipogenic marker expressions (PPAR-g2 and leptin) [15]. Collectively, it is reasonable to believe that PRFr represents an ideal molecular modulator for BMSCs osteogenesis. When applied concurrently in vivo, the cytokines originating from PRFr would exert stimulatory effects on BMSCS osteogenic gene and protein expressions thus accelerating new bone formation. However, one of the major concerns regarding the systemic administration of PRFr is the potential tumorigenic effects from the enriched growth factors despite their relatively short half-lives. Further studies need to be done in future to explore the biosafety of PRFr and to detect signs of tumorigenesis over the course of study.

In current study, osteoporosis was induced by bilateral OVX and bone mass attenuation was successfully demonstrated using micro-CT analysis (Fig.2). Compared to non-OVX mice, a significant reduction in BMD, BV/TV and Tb.N associated with an increased in Tb.Sp were observed in OVX mice at 8 weeks post-operatively, mimicking postmenopausal osteoporosis in women after estrogen withdrawal. Then, osteogenesis-promoting ability of BMSCs and PRFr was investigated in vivo using OVX mice exhibiting osteoporotic changes. Notably, single injection of either BMSCs, PRFr or their combination did not result in significant improvement of bony profile in treated mice compared to non-treated control. In contrast, an increasing trend of improved bony profile was observed in mice received quadruple injection of either PRFr, BMSCs or their combinatory use. However, only in mice which had received four times of systemic injection of combined BMSCs and PRFr showing significant improvement of BMD, BV/TV and Tb.N scores compared to non-treated OVX mice. In addition, this treatment regimen appeared to increase the formation of new trabecular bones evidenced by reduced trabecular separation shown in micro-CT and histology. The micro-CT data are quite consistent with the reports while BMSCs or adipose-derived stem cells were administered systemically to prevent bone loss in OVX animals [14, 28, 43, 44]. Moreover, these data also reinforced the notion that PRFr could improve the therapeutic efficacy of BMSC-based therapy for osteoporotic treatment. Additionally, the combined effect of PRFr and stem cell in enhancing osteogenesis is in line with other PRP-related in vivo studies. According to Wei and colleagues, PRP treatment could enhance bone microarchitecture and effectively upregulate osteogenic marker expressions in OVX-induced osteoporotic rats. In their animal model, the combinatory use of PRP and BMSCs significantly promotes healing of osteoporotic bone defects [44].

Our data confirmed the potential of PRFr in augmenting the effects of BMSCs in a preclinical trial with animal model. However, our study has several limitations. First, this preliminary study was conducted to evaluate the capacity of PRFr and BMSCs in promoting bony profile at 8 weeks after injections. However, it might be more thorough to observe the imaging and histological differences over different time points. Secondly, biomechanical analysis should be carried out in future the determine the difference in mechanical strength of analyzed bones among different experimental groups. Finally, histomorphometric analysis of interactions between osteoblasts and osteoclasts is lacking, thus further studies would be needed in future to have better understanding about the cellular mechanisms involved in this treatment strategy.

## Conclusion

In conclusion, we have demonstrated that systemic administration of combined BMSCs and PRFr protected against OVX-induced bone mass loss in mice. Moreover, the improvement of bony profile scores in quadruple injection group is better than the single-injection group, probably through the increase in effect size of cells and growth factors. Our data also revealed the combination therapy of BMSCs and PRFr was more potent in enhancing osteogenesis, which may provide insight for the development of a novel therapeutic strategy in osteoporosis treatment.

## Data Availability

The data analyzed during the current study are available from the corresponding author on reasonable request.

## Abbreviations

BMD: Bone marrow density
BMSC: Bone marrow stem cells
BV/TV: Bone volume versus total tissue volume
OVX: Ovariectomized
PRFr: Platelet-rich fibrin releasate
Tb.N: Trabecular number
Tb.Sp: Trabecular separation
